# Real-Time Decoder Architecture for LDPC–CPM

**DOI:** 10.3390/e27030255

**Published:** 2025-02-28

**Authors:** Erik Perrins

**Affiliations:** Department of Electrical Engineering and Computer Science, University of Kansas, Lawrence, KS 66049, USA; esp@ku.edu

**Keywords:** continuous phase modulation (CPM), low-density parity check (LDPC) code, channel coding, concatenated coding, iterative methods

## Abstract

This paper examines the iterative decoding of low-density parity check (LDPC) codes concatenated with continuous phase modulation (CPM). As relevant case studies, we focus on the family of three CPM waveforms that are embodied in the IRIG-106 aeronautical telemetry standard. Two of these CPMs have recently had LDPC codes designed for them for the first time, and thus the decoding complexity of these new schemes is of interest when considering adoption into the standard. We provide comprehensive numerical results that characterize the performance and iteration statistics of the joint LDPC–CPM decoder. These results identify the most advantageous decoder configurations and also expose a key design challenge, which is that LDPC-CPM decoders must deal with a large “peak to average” ratio in terms of global iterations. We show how a properly designed reference simulation can be used as a design tool to explore the performance of a large range of candidate systems without need for further simulation. We develop a real-time decoder architecture with fixed complexity and show how such a decoder can still achieve a relatively large maximum number of global iterations by introducing a trade-off between decoding latency and maximum global iterations. Our discussion shows that this scheme is generally applicable to LDPC-based schemes. We conclude with a comprehensive design study that demonstrates the accuracy of our methodology and its attractive performance–complexity trade-off.

## 1. Introduction

Continuous phase modulation (CPM) [1] is widely used in applications where miniaturization is of interest or strict size weight and power (SWaP) constraints are dominant. Aeronautical telemetry is one such an application and the three CPM waveforms found in the IRIG-106 standard [2] are the subject of study herein. The topic of forward error correction (FEC) codes for CPM has received regular attention over the years, e.g., [3,4,5,6,7,8,9,10]. Recently, in [11,12,13,14], a step-by-step and generally applicable procedure was developed for constructing capacity-approaching, protomatrix-based LDPC codes that can be matched to a desired CPM waveform. This development is significant for aeronautical telemetry because this design approach provides forward error correction solutions for two of the IRIG-106 CPM waveforms that were heretofore missing due to the generality of these waveforms.

In this paper, we build on the work in [13,14] by providing a solution to a key challenge faced by LDPC-based systems, which is that they must manage a large “peak to average” ratio when it comes to decoding iterations. The subject of LDPC decoder complexity reduction and iteration complexity has received a large amount of attention in the literature. In many studies, e.g., [15,16,17] and the references therein, numerous alternatives to the standard belief propagation (BP) decoder are explored, some of which have convergence and iteration advantages relative to the BP decoder. Other studies have developed real-time parallelization architectures that are well-suited for certain hardware, such as FPGAs [18] or GPUs [19]. In this paper, our focus is on iteration scheduling and prioritization that can take place *external* to a single LDPC iteration, thus any of the techniques just mentioned can be applied *internally* if desired. In [20], a dynamic iteration scheduling scheme similar to ours was introduced, although it ignored real-time considerations. We propose a flexible real-time decoder architecture whose complexity can be designed essentially to satisfy the average decoding requirements, which are relatively modest. We then introduce a design trade-off that allows a relatively large maximum number of global iterations to be achieved, when needed, in exchange for a fixed decoding latency.

We conduct a comprehensive study of the iteration statistics and frame error rate (FER) of the LDPC–CPM decoder, and we show how these statistics can be incorporated into a design procedure that accurately predicts FER performance as a function of maximum iterations. We confirm the accuracy of our approach with an in-depth design study of our real-time decoder architecture. Most importantly, our results demonstrate that our real-time decoder architecture can operate with modest complexity and with performance losses in the order of tenths of a dB.

The remainder of this paper is organized as follows. In Section 2, we lay out the system model and provide reference FER performance results. In Section 3, we highlight the essential differences between the recent LDPC–CPM schemes and traditional LDPC-based systems. In Section 4, we provide an in-depth discussion of the iteration statistics of LDPC–CPM decoders. In Section 5, we develop our real-time decoder architecture and we analyze its performance with a design study in Section 6, and in Section 7, we comment on the general applicability of our approach to LDPC-based systems. Finally, we offer concluding remarks in Section 8.

## 2. System Model

### 2.1. Transmitter Model

**Figure 1:**
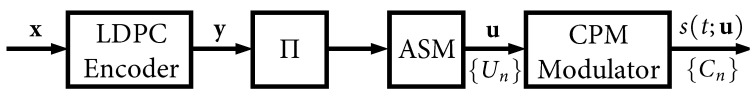
Transmitter model.

The LDPC–CPM transmission scheme is shown in Figure 1 and consists of an LDPC encoder and a CPM modulator that are separated by an interleaver (denoted by the symbol Π). For simplicity, our notation will assume the transmission of a single code word except in specific contexts where indexing is needed from one code word to the next. The information and code words (sequences) are denoted as $x≜{\left\{x_{i}\right\}}_{i=0}^{K-1}$ and $y≜{\left\{y_{i}\right\}}_{i=0}^{N-1}$, respectively, where R≜K/N is the code rate. We assume a *systematic* LDPC code; thus, having knowledge of y implies having knowledge of x, because the first *K* bits in y are the information bits, $y_{i}=x_{i}$, 0≤i≤K−1, while the remaining (N−K) bits are the redundant (parity) bits generated by the encoder. The interleaver permutes the order of the bits in the code word. The transmitter allows for the insertion of a known bit sequence called the *attached sync marker* (ASM) to help identify the beginning of each code word. The ASM for the next code word immediately follows, thus there is an ASM on either side of a given code word.

The transmitted word u is slightly longer than y (due to the ASM) and produces the transmitted CPM signal s(t;u). The bits in u are grouped into $n_{0}$-tuples (the CPM schemes in this paper use $n_{0}=1$ and $n_{0}=2$), and these $n_{0}$-tuples assume an *M*-ary alphabet, where $M=2^{n_{0}}$. The $n_{0}$-tuples can be expressed as individual bits or as symbols drawn from an integer alphabet, $U_{i}\in \left\{0,1,\ldots ,M-1\right\}$ (when $n_{0}=1$, the bit and symbol formats are the same). The CPM modulator can also be viewed as a continuous phase encoder (CPE), which has an *M*-ary input sequence $\left\{U_{n}\right\}$ and a non-binary output sequence $\left\{C_{n}\right\}$ [1,21]. The $\left\{U_{n}\right\}\to \left\{C_{n}\right\}$ viewpoint is also denoted in Figure 1. The CPE is a finite state machine, which can be demodulated/decoded using trellis processing. We define the following time intervals and energies: an information bit, $x_{i}$, has a duration of $T_{b}$ seconds and an energy of $E_{b}$; a coded bit, $y_{i}$, has a duration of $T_{c}=RT_{b}$ seconds and an energy of $E_{c}=RE_{b}$; a CPM symbol, $C_{n}$, has a duration of $T_{s}=n_{0}T_{c}=n_{0}RT_{b}$ seconds and an energy of $E_{s}=n_{0}E_{c}=n_{0}RE_{b}$ (both of which ignore the slight overhead of the ASM).

### 2.2. CPM Schemes

A full treatment of CPM can be found in [1]. A given CPM scheme (waveform) requires only that we specify (1) the size of its symbol alphabet, *M*, where each symbol has a duration of $T_{s}$ seconds; (2) its modulation index(es), $\left\{h_{i}\right\}$, of which there are $N_{h}$ in number and are constrained to be rational numbers of the form $h_{i}=k_{i}/p$, with *p* being the least common denominator; and (3) the shape of its frequency pulse, g(t), which is constrained to be non-zero only during the interval $\left[0,LT_{s}\right]$ and is normalized to have area of 1/2. The numbers of matched filters, $N_{MF}$, trellis states, $N_{S}$, and trellis edges (branches), $N_{E}$, respectively, are given by [1].

(1) $$N_{MF}=M^{L},\;\;\;\;\;N_{S}=pM^{L-1},\;\;\;\;\;N_{E}=pM^{L}=M\cdot N_{S}$$

The topic of complexity reduction for CPM has received much study [1]. In [22], a variety of complexity-reducing techniques were applied to one of the CPMs of interest in this paper (but with general applicability to other CPMs as well). Herein, the sole complexity-reducing technique we will draw from [22] is known as frequency pulse truncation (PT), which approximates the CPM signal at the receiver using a shortened value of *L*, i.e., $L_{Rx}<L$. With the PT approximation, the *transmitter* uses the original (exact) CPM model; however, the *demodulator/decoder* uses the smaller value of $L_{Rx}$ in place of *L*, which impacts (1).

**Figure 2:**
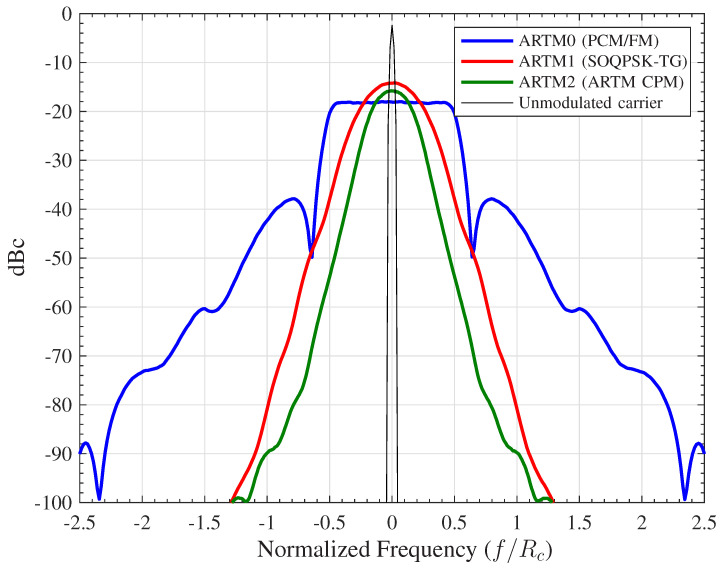
Power spectral densities (PSDs) of the three CPM schemes of interest in this paper. These are expressed in dB relative to an unmodulated carrier (dBc) and frequency normalized to the coded bit rate, $R_{c}=1/T_{c}$.

In this paper, we are interested in the three CPM schemes specified in the IRIG-106 standard [2] for use in aeronautical telemetry. The most recent two were developed in the early 2000s under the Advanced Range TeleMetry (ARTM) program in order to provide progressive “tiers” of spectral efficiency relative to the original/legacy CPM scheme. The power spectral densities (PSDs) of all three are shown in Figure 2. We define these three CPM waveforms as follows:

- **ARTM “Tier 0” (ARTM0)** is also known as PCM/FM. This is the least spectrally efficient CPM scheme we will study and has the following parameters (the long operational history of PCM/FM pre-dates modern digital transmitters, and thus the specification in IRIG-106 is written with analog transmitters in mind and allows for some variation (imprecision) in the modulation index and the frequency pulse. The current coded application presupposes a modern digital transmitter with an exact value of h=7/10 and a frequency pulse that is reasonably close to 2RC.): M=2, h=7/10, 2RC, which denotes a frequency pulse with a raised cosine (RC) shape with a duration of L=2. At the receiver, we will apply the PT approximation to model the signal as having $L_{Rx}=1$. This results in a demodulator that requires $N_{MF}=2$ MFs and a trellis with $N_{S}=10$ states and $N_{E}=20$ edges.
- **ARTM “Tier I” (ARTM1)** is also known as the telemetry group version of shaped offset quadrature phase shift keying (SOQPSK-TG). This has approximately twice (using the bandwidth definitions in [2]) the spectrum efficiency of ARTM0 and has the following parameters: M=2, h=1/2, 9TG, which denotes the L=9 frequency pulse shape obtained using the SOQPSK model developed in [23], and further discussed in [24]. At the receiver, we will apply the PT approximation to model the signal as having $L_{Rx}=2$. This results in a demodulator that requires $N_{MF}=4$ MFs and a trellis with $N_{S}=4$ states and $N_{E}=8$ edges. Because this binary waveform has h=1/2, it belongs to the family known as the minimum shift keying (MSK) type.
- **ARTM “Tier II” (ARTM2)** is also known as ARTM CPM [22]. This has approximately three times the spectrum efficiency of ARTM0 and has the following parameters: M=4, $\left\{h_{0},h_{1}\right\}=\left\{4/16,5/16\right\}$, 3RC. This scheme is notable for being non-binary (M=4, two bits per symbol) and being an example of multi-*h* CPM ($N_{h}=2$). At the receiver, we apply the PT approximation to model the signal as having $L_{Rx}=2$. This results in a demodulator that requires $N_{MF}=16$ MFs and a trellis with $N_{S}=64$ states and $N_{E}=256$ edges.

### 2.3. Receiver Model

**Figure 3:**
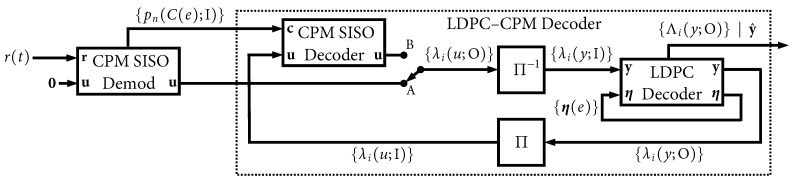
Receiver model.

The receiver is shown in Figure 3 has the dual task of CPM demodulation and LDPC decoding, which it accomplishes in an iterative fashion. The received signal model is

(2) $$r\left(t\right)=\sqrt{E_{s}/T_{s}}\;s\left(t;u\right)+w\left(t\right)$$

where w(t) is complex-valued additive white Gaussian noise (AWGN) with zero mean and power spectral density $N_{0}$.

For the binary variables u and y, the modules in Figure 3 exchange length-*N* packets (vectors) of *soft information* in the form of *L-values*. An L-value is defined as

(3) $$\lambda \left(u\right)=\ln\frac{P(u=1)}{P(u=0)}=p\left(u=1\right)-p\left(u=0\right)$$

where P(u=1) and P(u=0) are the two values of the probability mass function (PMF) belonging to a generic binary random variable *u*. If the PMF values already reside in the log domain, p(u=1)=lnP(u=1) and p(u=0)=lnP(u=0), then this “ratio” is simply the difference between the two terms. An L-value of zero indicates a 50–50 “tie” between a 0 and a 1. When no PMF is available, we use an L-value of zero (no information). A log likelihood ratio (LLR) is a well-known example of an L-value, but other examples include L-values belonging to an a priori PMF, an a posteriori PMF, or the extrinsic version of an a posteriori PMF. Input vectors in Figure 3 are denoted with “I” and contain a priori information, while output vectors are denoted with “O” and contain a posteriori information. In a situation where an output is later connected to another module’s input, the *extrinsic* version of such outputs is used.

The CPM processing in Figure 3 can be divided into a traditional CPM demodulator that is positioned *outside* the iterative decoding loop, and a CPM decoder that is located *inside* the iterative decoding loop. Both of these are responsible for generating a soft output for u, and thus they are labeled as soft-input soft-output (SISO) modules [25]. The CPM demodulator operates directly on r(t) and has no (zero) a priori information on its “**u**” input. The demodulator preserves the matched filter (MF) outputs it generates, and these describe a PMF belonging to the non-binary sequence $\left\{C_{n}\right\}$ (thus we cannot use L-values and must work with the entire log domain PMF). The CPE trellis is labeled with a unique value of this code symbol per edge of the trellis, C(e), and thus $\left\{p_{n}\left(C\left(e\right);I\right)\right\}$ denotes the length-$N_{E}$ PMF for $\left\{C_{n}\right\}$ at time index *n*. The entire size of $\left\{p_{n}\left(C\left(e\right);I\right)\right\}$ is length-$N_{E}$ times the number of time steps in the trellis, which is $N/n_{0}$, plus the possibility of a small number of trellis “warm up” time steps before and after the code word. The a priori u information for these warm up steps has 100% confidence because the ASM is known at the receiver, i.e., L-values of ±∞, where the polarity is determined by the values of the ASM bits that fall in the warm up intervals.

Once the CPM SISO demodulator has processed to the end of the received code word, its outputs are passed to the LDPC–CPM decoder, as shown in Figure 3. This joint decoder conducts a maximum of ${\mathrm{It}}_{\max}$ “global” iterations between the CPM and LDPC decoders, which are separated by an interleaver/deinterleaver. For the first global iteration, the CPM decoding task was completed externally by the demodulator, and that result was passed forward via the switch in position “A”. For successive global iterations, the switch is placed in position “B” so that the CPM SISO decoder’s updates are used.

Within the global loop, when it is the LDPC decoder’s turn to execute, it performs ${\mathrm{It}}_{\mathrm{loc}}$ iterations in a “local” loop. The internal state memory of the LDPC decoder is denoted as {η(e)}, which is a set of “edge memory buffers” (containing L-values) which have a total length equal to the number of edges in the Tanner graph of the LDPC code. These are initialized to zero at the start of the first global iteration and they are preserved and updated in each local LDPC iteration (they are simply preserved, but not updated, during the CPM portion of the global iteration). At the end of each global iteration, a parity check is performed on the decoded code word, $\hat{y}$, which arises from the full (non-extrinsic) a posteriori probability (APP) output of the LDPC decoder, $\left\{\Lambda_{i}\left(y;O\right)\right\}$. No further global iterations are needed if this parity check passes.

LDPC decoders are known to have a high “peak to average” ratio when it comes to the number of iterations needed in order to pass the parity check, cf. e.g., [26]. As with any setting where such a ratio is large, this can result in a system that is overdesigned and inefficient for average use (to accommodate the peak), or one that is underdesigned so that it performs poorly for peak use (but is efficient on average). The aim of this paper is to address this problem by developing a real-time decoder architecture with a fixed iteration budget that can achieve a (hopefully large) maximum value of ${\mathrm{It}}_{\max}$ iterations. We will use ${\mathrm{It}}_{\max}^{*}$ to denote an arbitrarily large value where further increases do not result in appreciable decreases in FER, and we select ${\mathrm{It}}_{\max}^{*}=512$ herein. At certain points in our development, we will consider performance as a function of ${\mathrm{It}}_{\max}$, where ${\mathrm{It}}_{\max}$ is viewed as an independent variable in the range $1\leq {\mathrm{It}}_{\max}\leq {\mathrm{It}}_{\max}^{*}$. And finally, our analysis will result in ${\bar{\mathrm{It}}}_{\max}$, which is the expected maximum value of global iterations that can be achieved in our real-time decoder.

### 2.4. LDPC Codes

In [13,14], a set of six LDPC codes were designed for ARTM0, ARTM1, and ARTM2 (18 unique codes in total). The dimensions and rates of these codes are listed in Table 1, along with additional values that will be discussed below. The study in [14] also explored the use of multiple local (LDPC-only) iterations per global iteration, i.e., ${\mathrm{It}}_{\mathrm{loc}}\geq 1$, where it was found that increasing ${\mathrm{It}}_{\mathrm{loc}}$ has the drawback of increasing the probability of undetected error (an undetected error occurs when the parity check passes but bit errors are present, i.e., the decoder output is a valid code word, but not the one that was transmitted) for the LDPC–CPM combinations considered both here and in [14]. The solution to this problem (i.e., the complete elimination of undetected errors) was two-fold: (1) a limited use of this feature, with the modest values of ${\mathrm{It}}_{\mathrm{loc}}={\mathrm{It}}_{\mathrm{loc}}^{*}$ that are listed in Table 1; and (2) a novel application of check node splitting and puncturing [27], which yields an equivalent parity check matrix, except that it has $N_{P}>N$ columns and, more importantly, has slightly different iterative behavior, such that it better avoids undetected errors. The benefit of using ${\mathrm{It}}_{\mathrm{loc}}\geq 1$ was also developed in [14], which is that it improves the execution speed of the joint iterative decoder. By adjusting ${\mathrm{It}}_{\mathrm{loc}}$, the computational burden of the LDPC half of the global iteration can be increased to better match that of the CPM half. When ${\mathrm{It}}_{\mathrm{loc}}\geq 1$, the LDPC decoder shoulders more of the work and fewer global global iterations are needed in order to pass the parity check. This is of particular interest for ARTM2, the SISO module, which is highly complex with its $N_{S}=64$ state trellis.

As discussed in [14], these are considerations that take place at the *receiver* only, with the *transmitter* being completely unaware and uninvolved. The transmitter always uses the original (non-punctured) generator matrix with $N=N_{\mathrm{NP}}$ columns, but the receiver uses one of the following:

1. **NP option**: the original (non-punctured) parity check matrix ($N_{\mathrm{NP}}$ columns) with ${\mathrm{It}}_{\mathrm{loc}}=1$;
2. **P option**: the punctured parity check matrix ($N_{P}$ columns) with ${\mathrm{It}}_{\mathrm{loc}}={\mathrm{It}}_{\mathrm{loc}}^{*}$.

This additional NP/P option brings the number of configurations considered in this paper to thirty-six: three modulation types, two block sizes, three code rates, and two puncturing/${\mathrm{It}}_{\mathrm{loc}}$ options.

Regardless of the puncturing option, the deinterleaver in Figure 3 always returns ${\left\{\lambda_{i}\left(y;I\right)\right\}}_{i=0}^{N_{\mathrm{NP}}-1}$, i.e., the data belonging only to the transmitted (non-punctured) bits. When puncturing is used, after deinterleaving takes place, the LDPC decoder input is zero-padded out to a length of $N_{P}$, i.e., ${\left\{\lambda_{i}\left(y;I\right)\right\}}_{i=N_{\mathrm{NP}}}^{N_{P}-1}=0$. The interleaver in Figure 3 always operates only on ${\left\{\lambda_{i}\left(y;O\right)\right\}}_{i=0}^{N_{\mathrm{NP}}-1}$ and ignores data belonging to punctured bits.

### 2.5. FER Performance

**Figure 4:**
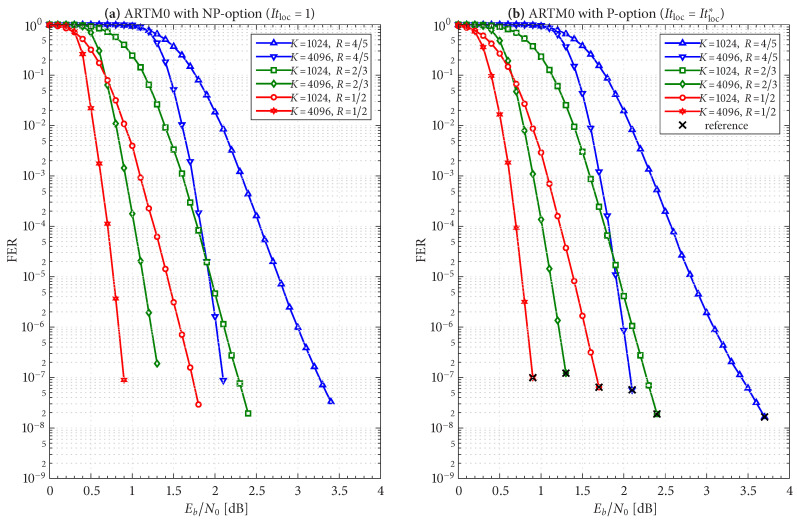
FER curves for the block sizes and code rates in Table 1 paired with ARTM0 (PCM/FM) with the (**a**) NP option (${\mathrm{It}}_{\mathrm{loc}}=1$) and (**b**) P option (${\mathrm{It}}_{\mathrm{loc}}={\mathrm{It}}_{\mathrm{loc}}^{*}$).

**Figure 5:**
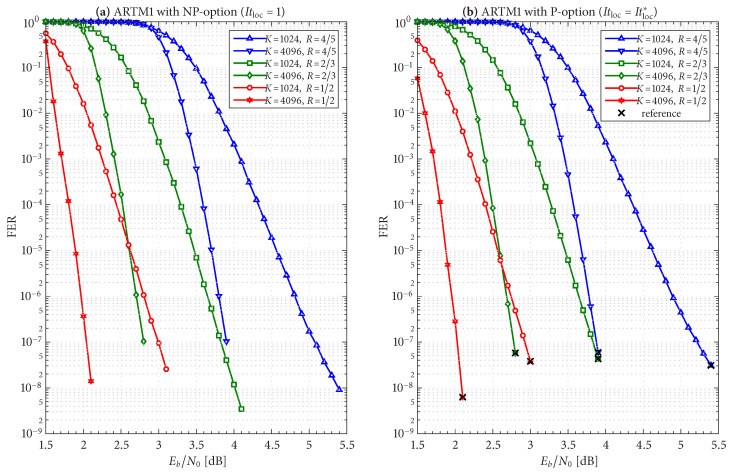
FER curves for the block sizes and code rates in Table 1 paired with ARTM1 (SOQPSK-TG) with the (**a**) NP option (${\mathrm{It}}_{\mathrm{loc}}=1$) and (**b**) P option (${\mathrm{It}}_{\mathrm{loc}}={\mathrm{It}}_{\mathrm{loc}}^{*}$).

**Figure 6:**
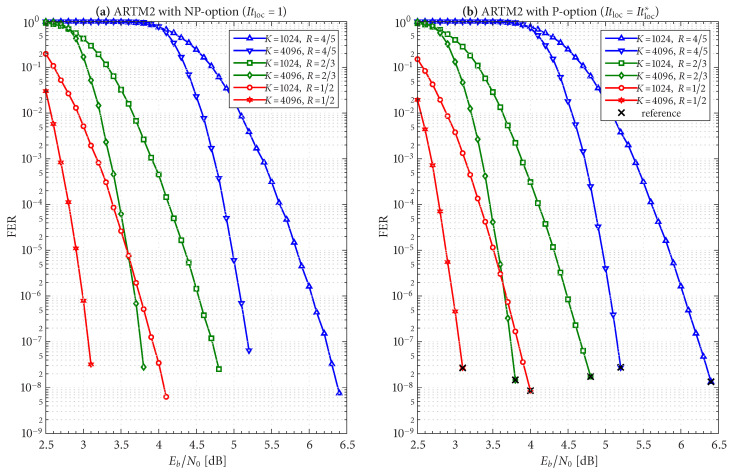
FER curves for the block sizes and code rates in Table 1 paired with ARTM2 (ARTM CPM) with the (**a**) NP option (${\mathrm{It}}_{\mathrm{loc}}=1$) and (**b**) P option (${\mathrm{It}}_{\mathrm{loc}}={\mathrm{It}}_{\mathrm{loc}}^{*}$).

The frame/code word error rate (FER) performance of the 36 configurations considered herein are shown in Figure 4, Figure 5 and Figure 6, where Figure 4 pertains to ARTM0 (PCM/FM), Figure 5 pertains to ARTM1 (SOQPSK-TG), and Figure 6 pertains to ARTM2 (ARTM CPM). These FER simulations were conducted using the high-speed, 16-bit, fixed-point, 8-way-parallelized configuration that was used in [14] (the execution speed of this architecture was also characterized in [14]). We selected ${\mathrm{It}}_{\max}^{*}=512$ for these simulations, but significantly fewer iterations are needed on average, as will be shown.

Generally speaking, the P option [sub-figure (b) in each of Figure 4, Figure 5 and Figure 6] has a slight FER advantage over the NP option [sub-figure (a) in each of Figure 4, Figure 5 and Figure 6] for all three modulations. This is attributable primarily to ${\mathrm{It}}_{\mathrm{loc}}={\mathrm{It}}_{\mathrm{loc}}^{*}\geq 1$, and this advantage was discussed in [14], along with the faster execution times enjoyed by ${\mathrm{It}}_{\mathrm{loc}}={\mathrm{It}}_{\mathrm{loc}}^{*}\geq 1$. The exceptions to this general rule are the K=1024, R=4/5 configurations (i.e., the shortest codes considered in this study), which have only ${\mathrm{It}}_{\mathrm{loc}}^{*}=1$. Although puncturing results in a slight FER degradation in these cases, it does completely resolve the relatively infrequent appearance of undetected errors. Thus, the P option receives our primary consideration from this point on due to its advantages with FER performance, execution speed, and the elimination of undetected errors.

The black reference “×” marks in sub-figure (b) in each of Figure 4, Figure 5 and Figure 6 identify an operating point for each configuration where the FER is in the order of $10^{-8}$ (with some variation due to the steep/shallow slope of the curves). These reference points are used in the iteration characterizations and design examples that follow.

## 3. Nonrecursive Transmitter and Receiver Model

In general, because CPMs are a family of nonlinear modulations with recursive memory, they must be treated separately from linear memoryless modulations, such as BPSK, QPSK, etc. However, for the important and special case of MSK-type CPMs, it is possible to take a nonrecursive viewpoint. A “precoder” (such as [28], Equation (7)) can be applied to MSK-type waveforms to “undo” the inherent CPM recursion, and this results in OQPSK-like behavior. From an information-theoretic perspective, this nonrecursive formulation allows MSK-type waveforms to be paired with LDPC codes that were designed for BPSK [5].

**Figure 7:**
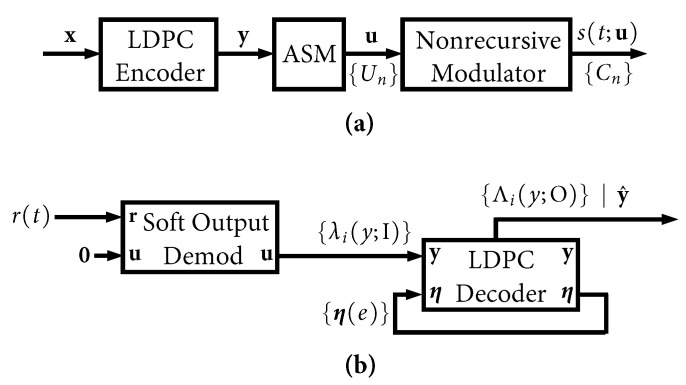
Nonrecursive AR4JA–ARTM1-NR models for (**a**) the transmitter and (**b**) the receiver.

This special case was exploited a decade ago in [8] to pair the AR4JA LDPC codes [27] with ARTM1-NR, and this became the first-ever FEC solution adopted into the IRIG-106 standard [2] for use in aeronautical telemetry. The transmitter and receiver models for this configuration are shown in Figure 7, which we refer to as ARTM1-NR. The transmitter model for AR4JA–ARTM1-NR in Figure 7a is identical to its recursive counterpart in Figure 1 except for the fact that there is no interleaver, and the nonrecursive modulator is used. However, the receiver model for AR4JA–ARTM1-NR is quite different. Because the nonrecursive waveform does not yield an extrinsic information “gain”, it is not included in the iterative decoding loop in Figure 7b. The sole purpose of the demodulator is to deliver a soft output (L-values) that can be used by the LDPC decoder without future updates (the soft output demodulator in Figure 7b is identical to the CPM SISO demodulator in Figure 3 aside from minor differences in the respective internal trellis diagrams and the elimination of the requirement to preserve $\left\{p_{n}\left(C\left(e\right);I\right)\right\}$ for future updates). Thus, the concept of global iterations is unnecessary, and the LDPC decoder iterates by itself up to a limit of ${\mathrm{It}}_{\max}$ iterations (or fewer if the parity check passes).

**Figure 8:**
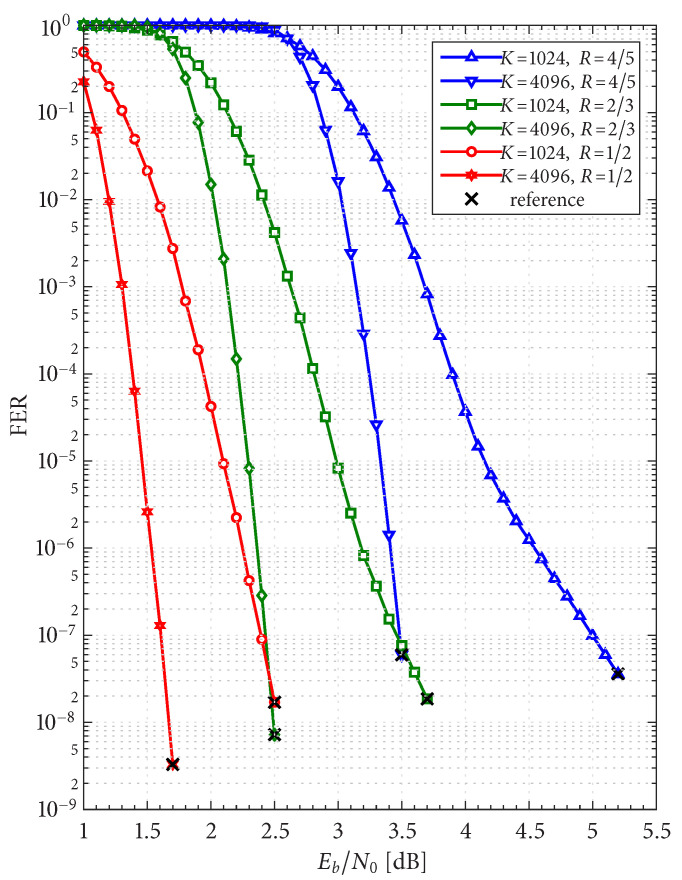
FER curves for the AR4JA–ARTM-NR codes.

The FER performance of the six AR4JA–ARTM-NR configurations is shown in Figure 8. While it is clear that the nonrecursive configurations (Figure 8) outperform the recursive configurations (Figure 5), as predicted by the analysis in [5], such an approach does not exist for non-MSK-type CPMs like ARTM0 and ARTM1, which underscores the merits of the LDPC–CPM codes we are studying.

Like any other LDPC-based system, the AR4JA–ARTM1-NR configuration suffers from the large “peak to average” ratio problem. Our numerical results will show that the AR4JA–ARTM1-NR decoder in Figure 7b typically requires more iterations than its LDPC-CPM counterparts, due to the fact that the LDPC decoder iterates by itself. However, the general behavior is the same, and thus it is compatible with the real-time decoder presented herein.

## 4. Characterization of the Iterative Behavior of the Receiver

As we have mentioned, LDPC decoders are known to have a high “peak to average” ratio when it comes to the number of iterations needed in order to pass the parity check, cf. e.g., [26]. We have defined ${\mathrm{It}}_{\max}$ above and we now define ${\mathrm{It}}_{\mathrm{avg}}$ as the average (i.e., *expected*) number of iterations needed to pass the parity check, which we will demonstrate shortly to be a function of $E_{b}/N_{0}$. We begin with an illustrative example.

**Figure 9:**
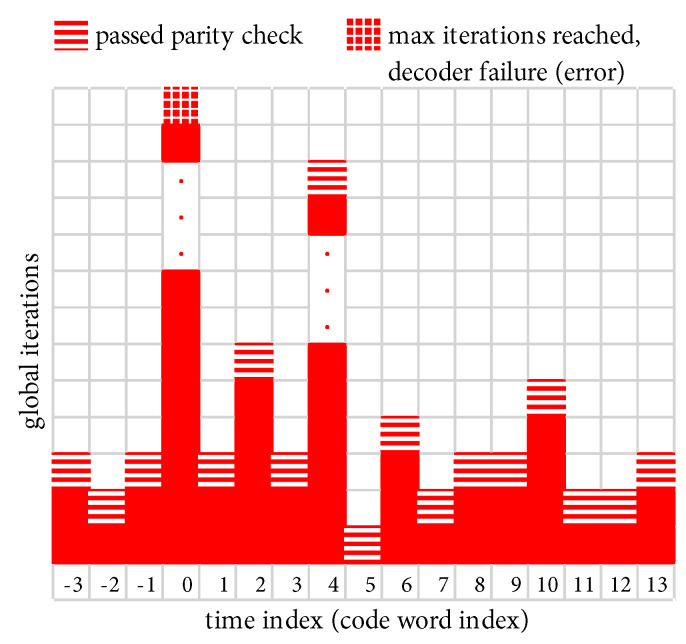
Illustration of the variation in global iterations needed per code word. In this example, the parity check typically passes after ${\mathrm{It}}_{\mathrm{avg}}=3$ global iterations. Two outliers are shown, at time indexes 0 and 4, both of which require a much larger number of iterations.

Figure 9 shows a scenario where $E_{b}/N_{0}$ is such that the parity check passes after a relatively small average of ${\mathrm{It}}_{\mathrm{avg}}=3$ global iterations. However, on rare occasions (two of which are shown in Figure 9), a received code word requires a large number of global iterations, which we refer to as an “outlier”. At time index 0 in Figure 9, the first outlier reaches ${\mathrm{It}}_{\max}$ without the parity check passing, which results in a decoder failure (frame error). At time index 4 in Figure 9, the second outlier eventually passes the parity check at an iteration count that is close to but less than ${\mathrm{It}}_{\max}$, which results in no frame error.

As $E_{b}/N_{0}$ increases, outliers become increasingly rare; however, the difference between a harmless outlier and an error event is determined by the choice of ${\mathrm{It}}_{\max}$. Thus, the low-FER performance becomes surprisingly sensitive to the value of ${\mathrm{It}}_{\max}$. In Figure 9, the FER would double if ${\mathrm{It}}_{\max}$ were set to a slightly smaller value (a FER penalty of 2×), because both outliers would result in a decoder failure. We now develop a simple means of quantifying the FER penalty under the “what if” scenario of varying ${\mathrm{It}}_{\max}$. We begin by characterizing the behavior of ${\mathrm{It}}_{\mathrm{avg}}$.

### 4.1. Average Global Iterations per Code Word

**Figure 10:**
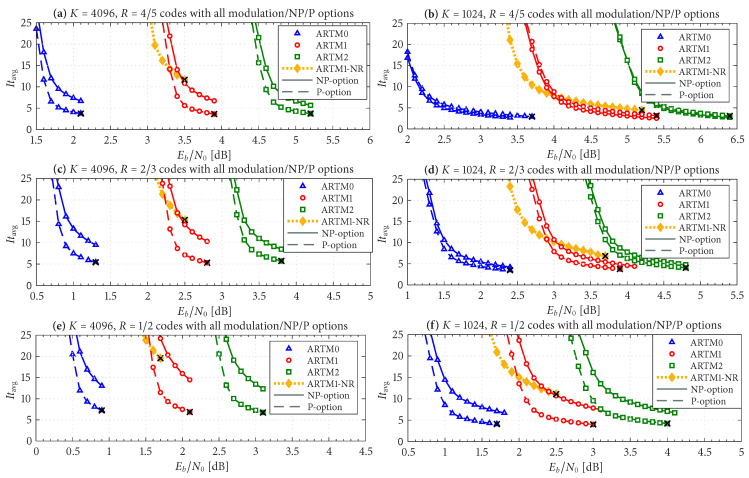
${\mathrm{It}}_{\mathrm{avg}}$ for the six different combinations of block size and code rate, as listed in the title of each sub-figure. Curves for the NP/P options are displayed in each sub-figure for easy comparison. The values marked with a black “×” are used as reference points later in this paper.

Figure 10 shows ${\mathrm{It}}_{\mathrm{avg}}$ for all 36 LDPC–CPM design configurations, plus the 6 AR4JA–ARTM1-NR configurations, which is demonstrated to decrease monotonically as $E_{b}/N_{0}$ increases (i.e., as FER decreases). The curves are grouped according to block size and code rate, i.e., the K=4096, R=4/5 configurations of ARTM0, ARTM1, ARTM2, and ARTM1-NR are plotted together in Figure 10a, and so forth. In each sub-figure, there is a side-by-side comparison of the NP/P options, where we see that using ${\mathrm{It}}_{\mathrm{loc}}={\mathrm{It}}_{\mathrm{loc}}^{*}$ (P option) can cut ${\mathrm{It}}_{\mathrm{avg}}$ by as much as a half. The motivation for grouping by block size and code rate is that the values of ${\mathrm{It}}_{\mathrm{avg}}$ are comparable across all three modulation types when the code rate, block size, *and* (surprisingly) FER are held constant. For example, with the K=4096, R=1/2 codes [Figure 10e] for ARTM0, ARTM1, and ARTM2, respectively, we observed ${\mathrm{It}}_{\mathrm{avg}}=\left\{13.0,14.5,12.5\right\}$ when operating with an FER in the order of $10^{-8}$ under the NP option. At this same FER operating point for the K=1024, R=4/5 codes [Figure 10b], we observed ${\mathrm{It}}_{\mathrm{avg}}=\left\{2.70,2.75,2.80\right\}$ for the respective modulations under the NP option. In all cases, the ARTM1-NR configurations (orange curves) result in larger values of ${\mathrm{It}}_{\mathrm{avg}}$, and in some cases, many times the respective P option (red curves).

### 4.2. Global Iteration Histogram, Shortened Histograms, and FER Penalty Factor

In order to characterize the “peak” iterations, we configure the receiver to maintain a histogram of the global iterations needed to pass the parity check, which is straightforward to implement. We denote this histogram as a length (${\mathrm{It}}_{\max}+1)$ sequence of points where the final value (called the *endpoint*) is $E[{\mathrm{It}}_{\max}+1]$ and the remaining values are {N[It]}, $1\leq \mathrm{It}\leq {\mathrm{It}}_{\max}$. N[It] stores the frequency (i.e., *count*) of observed instances that needed exactly It global iterations to pass the parity check. It is understood that the endpoint, $E[{\mathrm{It}}_{\max}+1]$, contains the frequency (count) of observed decoder failures (frame errors), i.e., instances where the parity check did not pass after ${\mathrm{It}}_{\max}$ global iterations. The total number of code words observed is

(4) $$N_{\mathrm{tot}}=E\left[{\mathrm{It}}_{\max}+1\right]+\sum_{\mathrm{It}=1}^{{\mathrm{It}}_{\max}}N\left[\mathrm{It}\right]$$

which can be used to normalize the histogram, yielding an empirical PMF that, in turn, can be used to compute ${\mathrm{It}}_{\mathrm{avg}}$ (i.e., the expected value), and so forth. The FER is simply $E\left[{\mathrm{It}}_{\max}+1\right]/N_{\mathrm{tot}}$. A long *reference* histogram of length (${\mathrm{It}}_{\max}^{*}+1)$ can be *shortened* to a histogram (${\mathrm{It}}_{\max}+1)$ in length, where ${\mathrm{It}}_{\max}\leq {\mathrm{It}}_{\max}^{*}$, simply by taking the points in the reference histogram that exceed ${\mathrm{It}}_{\max}$ and *marginalizing* them into the shortened endpoint:

(5) $$E\left[{\mathrm{It}}_{\max}+1\right]=E\left[{\mathrm{It}}_{\max}^{*}+1\right]+\sum_{\mathrm{It}={\mathrm{It}}_{\max}+1}^{{\mathrm{It}}_{\max}^{*}}N\left[\mathrm{It}\right]$$

The value of $N_{\mathrm{tot}}$ is the same for the reference histogram and any shortened histogram derived from it. The shortened endpoint in (5) can be viewed as a function (sequence) in its own right, with ${\mathrm{It}}_{\max}$ serving as the independent variable in the range $1\leq {\mathrm{It}}_{\max}\leq {\mathrm{It}}_{\max}^{*}$; its shape is similar to a complementary cumulative distribution function (CCDF).

**Figure 11:**
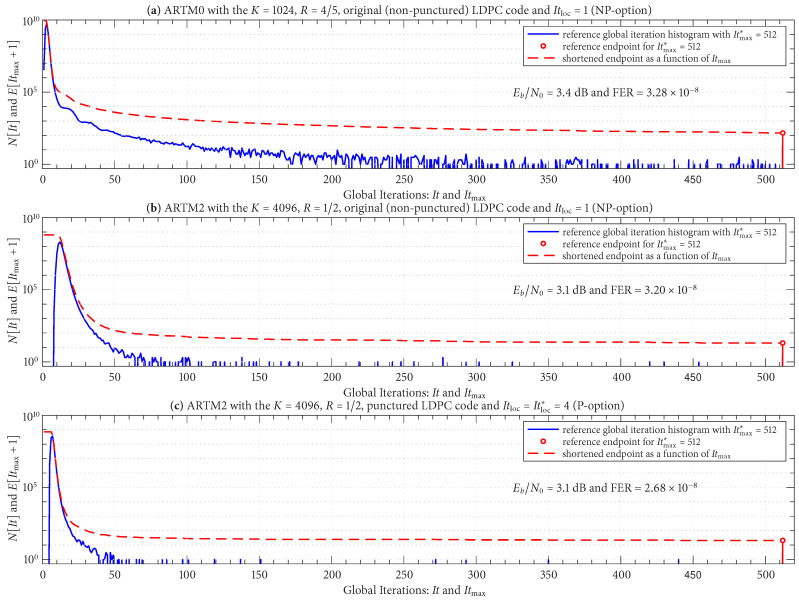
Reference global iteration histograms and shortened endpoint functions for three representative configurations, as listed in the title of each sub-figure.

Figure 11 shows reference global iteration histograms (plotted on a log scale) for three LDPC–CPM configurations; the details of each configuration are listed in the title of each sub-figure. The $E_{b}/N_{0}$ operating points are also stated and were chosen such that the FER is in the order of $10^{-8}$ using a reference value of ${\mathrm{It}}_{\max}^{*}=512$ global iterations. The reference endpoints are shown as red “stems” at the far right (positioned at ${\mathrm{It}}_{\max}^{*}+1=513$). The log scale gives the reference endpoints a relatively noticeable amplitude; nevertheless, it is the case that $E\left[{\mathrm{It}}_{\max}^{*}+1\right]/N_{\mathrm{tot}}$ for each configuration is in the order of $10^{-8}$. Figure 11 also shows the shortened endpoint in (5) as a function of ${\mathrm{It}}_{\max}$ using a red line.

The configuration in Figure 11a demonstrates that the histogram can have a heavy “upper tail”, which translates to a need for a relatively large ${\mathrm{It}}_{\max}$ in such cases. The configurations in Figure 11b,c are identical to each other, except (b) uses an original (non-punctured) code with ${\mathrm{It}}_{\mathrm{loc}}=1$ (NP option) and (c) uses a punctured code with ${\mathrm{It}}_{\mathrm{loc}}={\mathrm{It}}_{\mathrm{loc}}^{*}=4$ (P option). The impact of ${\mathrm{It}}_{\mathrm{loc}}={\mathrm{It}}_{\mathrm{loc}}^{*}=4$ is very noticeable in Figure 11c and results in a distribution with a lower mean [confirmed by Figure 10e], a lower variance, and an upper tail that is significantly less heavy.

Because this study entails 36 configurations, we consider Figure 11 to be representative of general trends and omit repetitive results for the other configurations. However, as has already been foreshadowed by the discussion above, the red stems in Figure 11 represent the FER of the given reference configuration (when normalized by $N_{\mathrm{tot}}$), and thus the red traces in Figure 11 represent the variation (penalty) in FER that can be expected under the “what if” scenario of changing the value of ${\mathrm{It}}_{\max}$. We formalize this relationship by taking $E\left[{\mathrm{It}}_{\max}+1\right]/N_{\mathrm{tot}}$ (the FER as a function of ${\mathrm{It}}_{\max}$) and normalizing it by $E\left[{\mathrm{It}}_{\max}^{*}+1\right]/N_{\mathrm{tot}}$ (the reference FER):

(6) $$X_{{\mathrm{It}}_{\max}^{*}}\left[{\mathrm{It}}_{\max}\right]=\frac{E\left[{\mathrm{It}}_{\max}+1\right]/N_{\mathrm{tot}}}{E\left[{\mathrm{It}}_{\max}^{*}+1\right]/N_{\mathrm{tot}}}=\frac{E[{\mathrm{It}}_{\max}+1]}{E[{\mathrm{It}}_{\max}^{*}+1]}$$

which we refer to as the FER *penalty factor*.

**Figure 12:**
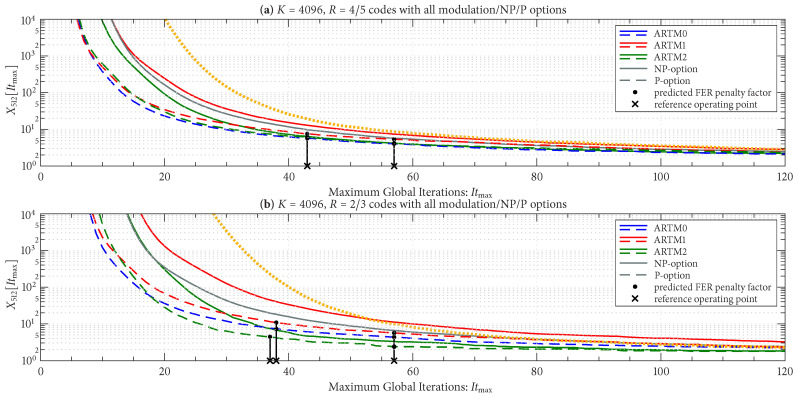

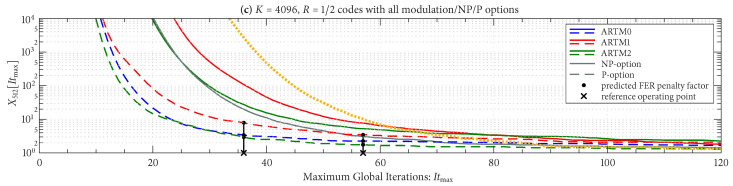
FER penalty factor, $X_{512}\left[{\mathrm{It}}_{\max}\right]$, for the K=4096 codes with (**a**) R=4/5, (**b**) R=2/3, and (**c**) R=1/2. Curves for the NP/P options are displayed in each sub-figure for easy comparison.

**Figure 13:**
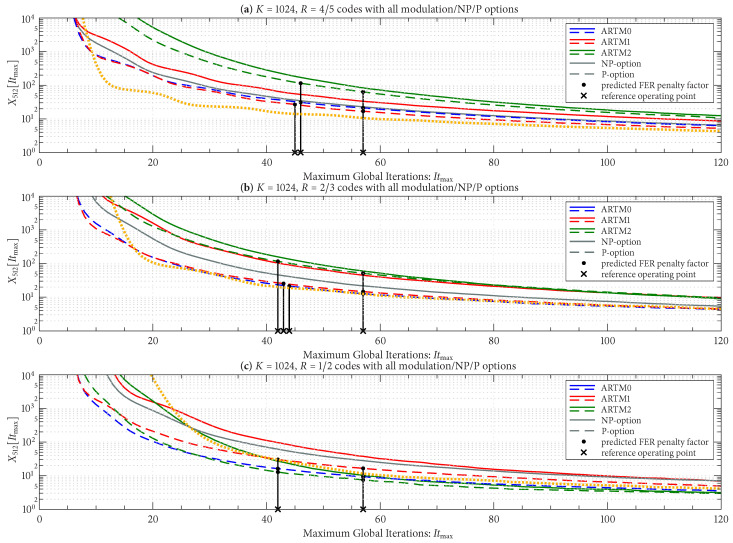
FER penalty factor, $X_{512}\left[{\mathrm{It}}_{\max}\right]$, for the K=1024 codes with (**a**) R=4/5, (**b**) R=2/3, and (**c**) R=1/2. Curves for the NP/P options are displayed in each sub-figure for easy comparison.

As with ${\mathrm{It}}_{\mathrm{avg}}$, we group the curves for $X_{512}\left[{\mathrm{It}}_{\max}\right]$ according to block size and code rate (six groupings), with the FER operating in the order of $10^{-8}$. Figure 12 shows $X_{512}\left[{\mathrm{It}}_{\max}\right]$ for the three groupings with the longer block length of K=4096, and Figure 13 does the same for the three groupings with the shorter block length of K=1024, as listed in the title of each sub-figure. There is a side-by-side comparison in each sub-figure of the NP/P options, where we see that the P option has a smaller FER penalty factor for a given ${\mathrm{It}}_{\max}$ in all cases. The black vertical lines (bars) in Figure 12 and Figure 13 pertain to the ${\mathrm{It}}_{\max}$ design procedure and will be explained shortly.

Figure 12 and Figure 13 also show the FER penalty factor for the AR4JA-ARTM1-NR configurations (orange curves). In most cases, AR4JA-ARTM1-NR requires larger values of ${\mathrm{It}}_{\max}$ to achieve a fixed FER penalty factor (compare the orange and red curves).

## 5. Real-Time Decoder with a Fixed Iteration Budget

### 5.1. Architecture

We now develop a relatively simple architecture that addresses the high “peak to average” problem with global iterations. The main idea is that if the hardware can accomplish only a limited and fixed “budget” of ${\mathrm{It}}_{\mathrm{bgt}}$ global iterations during a single code word interval, it is better to distribute these iterations over many code words simultaneously than it is to devote them solely to the current code word. The architecture does not seek to reduce the peak-to-average ratio. Instead, the architecture seeks to focus the hardware complexity on the *average* decoding requirement. The mechanism that addresses the peak requirement is the introduction of decoding delay (latency). The two primary “costs” of this approach are thus *memory* (storage) and *latency* (delay). The *processing complexity* is held constant (fixed) and the primary design consideration is the trade-off that is introduced between maximum iterations (${\mathrm{It}}_{\max}$) and latency.

**Figure 14:**
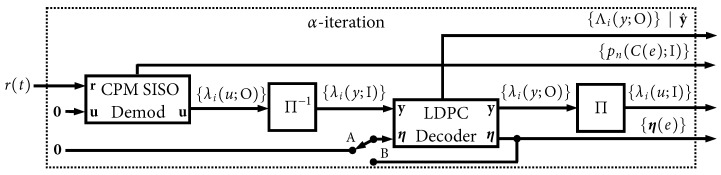
Graphical depiction of the α-iteration, which is the first global iteration.

We begin by unraveling the global iterative decoding loop in Figure 3. A block diagram of the *first* global iteration is shown in Figure 14, which we refer to as the α-iteration. The sole input to this iteration is the signal received from the AWGN channel, r(t) in (2), and all other a priori inputs are initialized to zero (no information). This is the only instance where r(t) is used, and, likewise, the only instance where the more-complex CPM *demodulator* SISO is used. This iteration produces four outputs. The first is the final output of the LDPC decoder, which is the soft MAP output, $\lambda_{i}\left(y;O\right)$, and the pass/fail parity check result. The three other outputs are intermediate results/state variables that will be used in subsequent iterations: $\left\{p_{n}\left(C\left(e\right);I\right)\right\}$, $\left\{\lambda_{i}\left(u;I\right)\right\}$, and {η(e)}.

**Figure 15:**
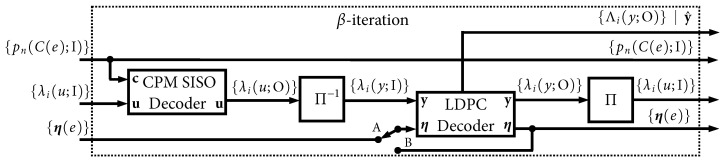
Graphical depiction of a β-iteration, which is used for all iterations after the first.

Figure 15 shows the processing that takes place in all iterations after the first, which we refer to as β-iterations. These iterations accept the three intermediate results/state variables from a previous iteration and produce the same four outputs that were discussed above. The less-complex CPM *decoder* SISO is used in the β-iterations. The notation in Figure 14 and Figure 15 highlights the “local” iterative loop that is used when ${\mathrm{It}}_{\mathrm{loc}}\geq 1$. It is worth pointing out that several parallelization strategies were developed in [14] that were shown to be very effective in speeding up these iterations.

**Figure 16:**
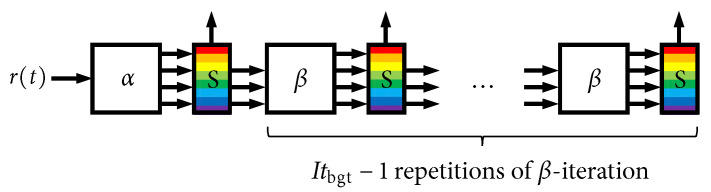
Global iterative chain with a fixed length (complexity) of ${\mathrm{It}}_{\mathrm{bgt}}$ iterations.

Figure 16 shows how Figure 14 and Figure 15 are connected together to form a “chain” with a fixed length of ${\mathrm{It}}_{\mathrm{bgt}}$ iterations. There is a rainbow-colored scheduling block (“S”) situated between each iteration. This block executes a scheduling algorithm and has storage for a set of *decoder buffers*, B[b], $0\leq b\leq N_{\mathrm{buf}}-1$, where $N_{\mathrm{buf}}$ is the number of such buffers; the many colors pictured within the S-block are meant to depict each individual decoder buffer. These data structures contain all state variables that are needed to handle the decoding of a single code word from one global iteration to the next, such as the following:

- Storage for $\lambda_{i}\left(y;O\right)$ and the pass/fail parity check result;
- Storage for $\left\{p_{n}\left(C\left(e\right);I\right)\right\}$;
- Storage for $\left\{\lambda_{i}\left(u;I\right)\right\}$;
- Storage for {η(e)};
- A flag to indicate if the buffer is “active” or not;
- An integer index indicating the sequence order of its assigned code word;
- A counter indicating the current latency of the assigned code word (i.e., the number of time steps the buffer has been active);
- The number of global iterations the buffer has experienced so far (for statistical purposes);

Because each β-iteration involves the state variables contained in a given B[b], it is thus trivial for the scheduling block to swap in/out a pointer to different B[b] from one β-iteration to the next. Using the parallelization strategies in [14], the entire chain of β-iterations can be executed quite rapidly, i.e., it is likely the case that the entire chain of β-iterations can be executed in the same amount of time as one α-iteration. A given buffer is allowed to be active for maximum latency (count) of $L_{\max}$ time steps (i.e., complete executions of the fixed-length chain in Figure 16), at which point a decoder failure (frame error) is declared. We have introduced $N_{\mathrm{buf}}$ and $L_{\max}$ as distinct parameters; however, going forward, we will assume that the amount decoder memory is the same as the maximum latency, i.e., $N_{\mathrm{buf}}=L_{\max}$.

### 5.2. Scheduling Scheme with β-Mode = TRUE

**Figure 17:**
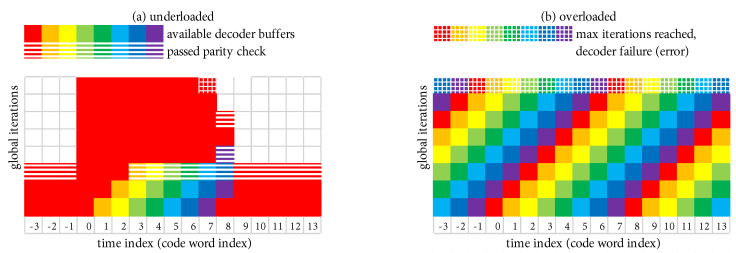
Example system with ${\mathrm{It}}_{\mathrm{bgt}}=8$ and $L_{\max}=8$ with β-mode = TRUE: (**a**) underloaded (${\mathrm{It}}_{\mathrm{avg}}<{\mathrm{It}}_{\mathrm{bgt}}$), operating conditions are favorable with ${\mathrm{It}}_{\mathrm{avg}}=3$ and an outlier can receive ${\bar{\mathrm{It}}}_{\max}=46$ iterations; (**b**) overloaded (${\mathrm{It}}_{\mathrm{avg}}>{\mathrm{It}}_{\mathrm{bgt}}$), operating conditions are poor and the system produces a frame error each time due to ${\mathrm{It}}_{\mathrm{avg}}>{\mathrm{It}}_{\mathrm{bgt}}$.

Figure 17a,b show the execution of a simple example scheduling scheme that is operating, respectively, in *underloaded* and *overloaded* conditions. The example scheduling scheme consists of the system in Figure 16 configured to perform a total of ${\mathrm{It}}_{\mathrm{bgt}}=8$ global iterations during each time index, where the scheduling block has storage for $N_{\mathrm{buf}}=8$ decoder buffers, and the maximum allowable decoding latency is $L_{\max}=8$ code words. The simple scheduling strategy is that each active decoder buffer receives one iteration at each time index, after which the excess/unused iterations are given to the “oldest” code word. At least one decoder buffer must be inactive at the beginning of each time index in order to receive the output of the α-iteration. We refer to this scheduling strategy as β-mode = TRUE because each active buffer receives at least one iteration each time (this terminology will be given more context shortly).

Under the favorable conditions present in Figure 17a, $E_{b}/N_{0}$ is such that ${\mathrm{It}}_{\mathrm{avg}}<{\mathrm{It}}_{\mathrm{bgt}}$ (in fact, ${\mathrm{It}}_{\mathrm{avg}}=3$), which results in the decoder being underloaded on average. The code word at time index 0 is an “outlier”. During the window of length $L_{\max}=8$ where the outlier is allowed to be active (time indexes 0–7), three additional decoder buffers are activated to handle the new code words as they arrive. During this window, after each active decoder receives its single iteration, the excess/unused iterations are given to the outlier. This permits the outlier to receive a total of 46 global iterations until a decoder failure (frame error) occurs when $L_{\max}=8$ is reached at time index 7 (i.e., there are exactly 46 red-shaded squares during time indexes 0–7). Once this occurs, the system quickly “catches up” and returns to a state where only one decoder buffer is active. We emphasize the fact that the processing complexity remains fixed at ${\mathrm{It}}_{\mathrm{bgt}}=8$ iterations per time step.

Under the poor conditions present in Figure 17b, $E_{b}/N_{0}$ is such that ${\mathrm{It}}_{\mathrm{avg}}>{\mathrm{It}}_{\mathrm{bgt}}$, which results in the decoder being overloaded on average. Each code word is an outlier in the sense that they all require more iterations than can be budgeted. All $N_{\mathrm{buf}}=8$ decoder buffers are put to use to handle the new code words as they arrive. The “oldest” decoder buffer is forced to fail when $L_{\max}=8$ is reached after it has received only ${\mathrm{It}}_{\mathrm{bgt}}=8$ global iterations.

Algorithm 1 provides pseudocode for the simple scheduling scheme we have used in the above examples. The receiver maintains a master time (sequence) index, *k*, for the code words as they arrive. When the *k*-th code word arrives, we assume that at least one decoder buffer is inactive and available for the α-iteration, which is identified by the index $b_{\alpha }$. When the decoding iterations are completed, the receiver outputs the code word $\hat{y}\left[k-L_{\max}+1\right]$, which has a fixed latency (delay) of $L_{\max}-1$ code words. Therefore, the receiver always commences a decoding operation each time a code word arrives and it always outputs a decoded code word. The following variables are required by the algorithm and are updated as needed: $C_{\max}$ is the maximum latency count of any active buffer and $b_{\max}$ is the index of this buffer, where $b_{\max}=-1$ indicates there are no buffers currently active; and $b_{\mathrm{inactive}}$ is the index of a buffer that is currently inactive.

**Algorithm 1 **Example real-time global iteration scheduling scheme.
  1:**Input:** Received signal r(t) belonging to the *k*-th LDPC code word in a transmitted sequence.  2:**Assumptions:** *k* is the master time (sequence) index, $b_{\alpha }$ indicates an inactive buffer, β-mode is defined.  3:**Initialization:** Increment *k*, activate $B[b_{\alpha }]$, and set ${\mathrm{It}}_{\mathrm{cur}}=0$.
**Perform the α-iteration and possibly one β-iteration per active buffer:**
  4:
**for** $b=0,1,\ldots ,N_{\mathrm{buf}}-1$ **do**
  5:      **if** B[b] is Active **then**  6:              Increment B[b]’s latency counter;  7:            **if** $b==b_{\alpha }$ **then**  8:               Perform the α-iteration using r(t) and filling B[b];  9:               Increment ${\mathrm{It}}_{\mathrm{cur}}$;  increment B[b]’s iteration counter;  deactivate B[b] if parity check passes;10:            **else if** β-mode == TRUE **then**11:               Perform a β-iteration drawing from B[b] and updating B[b];12:               Increment ${\mathrm{It}}_{\mathrm{cur}}$;  increment B[b]’s iteration counter;  deactivate B[b] if parity check passes;13:            **end if**14:      **end if**15:
**end for**

**Allocate Remaining β-Iterations to Oldest Buffer:**
16:**for** $i={\mathrm{It}}_{\mathrm{cur}},\ldots ,{\mathrm{It}}_{\mathrm{bgt}}-1$ **do**17:      Identify $b_{\max}$;18:      **if** $b_{\max}==-1$ **then**19:            Stop iterations;20:      **else**21:            Perform a β-iteration using $B[b_{\max}]$;22:            Increment ${\mathrm{It}}_{\mathrm{cur}}$;  increment $B[b_{\max}]$’s iteration counter;  deactivate $B[b_{\max}]$ if parity check passes;23:      **end if**24:
**end for**

**Ensure at Least One Buffer is Available for Next Time:**
25:Identify $C_{\max}$, $b_{\max}$, and $b_{\mathrm{inactive}}$;26:**if** $C_{\max}==L_{\max}$ **then**27:      Declare a *decoder failure*;28:      Deactivate $B[b_{\max}]$;29:      Set $b_{\alpha }=b_{\max}$;30:
**else**
31:      Set $b_{\alpha }=b_{\mathrm{inactive}}$;32:
**end if**
33:Output $\hat{y}\left[k-L_{\max}+1\right]$, which was filled somewhere above when its buffer was deactivated;


The process of “activating” a decoder buffer for the α-iteration consists of initializing the $\left\{\lambda_{i}\left(u;I\right)\right\}$ and {η(e)} arrays to zero; setting the active flag; resetting the latency and iteration counters to zero; and saving the master sequence index *k*.

The process of “deactivating” a decoder buffer consists of copying $\hat{y}$ from the internal memory of the buffer to the receiver output stream, in proper sequence order, as indicated by the stored sequence index *k*; likewise, copying the accompanying parity check result and global iteration count in their proper sequence orders; and clearing the active flag.

### 5.3. Scheduling Scheme with β-Mode = FALSE

We now consider a variation of this example scheduling scheme, where the α-iteration takes place as before, but *all*β-iterations are reserved only for the “oldest” code word. Because β-iterations are withheld from the other active buffers, we refer to this variation as β-mode = FALSE. The pseudocode in Algorithm 1 includes the notation necessary for β-mode = TRUE/FALSE.

**Figure 18:**
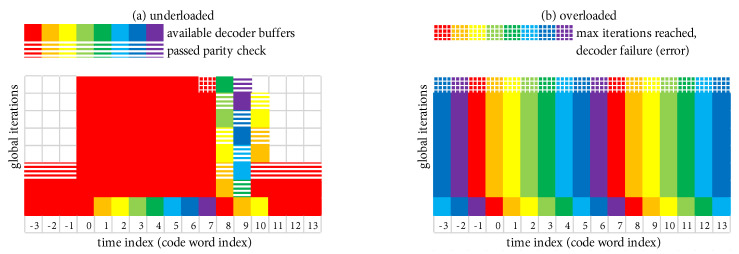
Example system with ${\mathrm{It}}_{\mathrm{bgt}}=8$ and $L_{\max}=8$ with β-mode = FALSE: (**a**) underloaded (${\mathrm{It}}_{\mathrm{avg}}<{\mathrm{It}}_{\mathrm{bgt}}$), operating conditions are favorable with ${\mathrm{It}}_{\mathrm{avg}}=3$, and an outlier can receive ${\bar{\mathrm{It}}}_{\max}=57$ iterations; (**b**) overloaded (${\mathrm{It}}_{\mathrm{avg}}>{\mathrm{It}}_{\mathrm{bgt}}$), operating conditions are poor and the system produces a frame error each time due to ${\mathrm{It}}_{\mathrm{avg}}>{\mathrm{It}}_{\mathrm{bgt}}$.

Figure 18a,b show the execution of β-mode = FALSE, respectively, in *underloaded* and *overloaded* conditions. We select ${\mathrm{It}}_{\mathrm{bgt}}=8$ and $L_{\max}=8$ as before. When overloaded with β-mode = FALSE in Figure 18b, each code word receives only ${\mathrm{It}}_{\mathrm{bgt}}=8$ global iterations, as was the case with β-mode = TRUE.

When β-mode = FALSE is underloaded in Figure 18a, the outlier at time index 0 is able to receive a total of 57 global iterations before $L_{\max}=8$ is reached at time index 7 (i.e., there are exactly 57 red-shaded squares during time indexes 0–7); this is an increase of 11 global iterations over β-mode = TRUE. Although the decoder has fallen further behind with β-mode = FALSE, the first code word that must be decoded in the “catch up” phase is still allowed its full budget of ${\mathrm{It}}_{\mathrm{bgt}}=8$ global iterations. Using Bayes’ rule [P(A∩B)=P(A)·P(B|A)], the probability of the joint event of an outlier exceeding ${\mathrm{It}}_{\max}$ iterations, immediately followed by a code word exceeding ${\mathrm{It}}_{\mathrm{bgt}}$ iterations, is $E\left[{\mathrm{It}}_{\max}+1\right]/N_{\mathrm{tot}}\cdot E\left[{\mathrm{It}}_{\mathrm{bgt}}+1\right]/N_{\mathrm{tot}}$. Thus, the probability of an outlier error (i.e., the original FER) plus the probability that such an error is immediately followed by a second error is $E\left[{\mathrm{It}}_{\max}+1\right]/N_{\mathrm{tot}}\cdot \left(1+E\left[{\mathrm{It}}_{\mathrm{bgt}}+1\right]/N_{\mathrm{tot}}\right)$. The benefit of additional global iterations (11 in our example) is embodied in a reduced value of $E\left[{\mathrm{It}}_{\max}+1\right]/N_{\mathrm{tot}}$. The disadvantage is embodied in the potential FER increase due to the factor $\left(1+E\left[{\mathrm{It}}_{\mathrm{bgt}}+1\right]/N_{\mathrm{tot}}\right)$. In our numerical results in Section 6, we will show that the benefit of β-mode = FALSE outweighs the disadvantage.

### 5.4. Expected Minimum and Maximum Iterations of Example Scheduling Scheme

As discussed above, when the system is overloaded, i.e., ${\mathrm{It}}_{\mathrm{avg}}>{\mathrm{It}}_{\mathrm{bgt}}$, each code word will receive at least a minimum—and likely no more than a maximum—of ${\mathrm{It}}_{\mathrm{bgt}}$ iterations. This is because it is highly unlikely that a significant number of unused iterations will become available for use by other code words.

The probability that the system is overloaded ($P_{\mathrm{ov}}$) is of great interest, but this probability is difficult to quantify in precise terms. However, in our extensive numerical results, we have observed $P_{\mathrm{ov}}\approx 1$ when ${\mathrm{It}}_{\mathrm{avg}}$ exceeds ${\mathrm{It}}_{\mathrm{bgt}}$, and we have also observed $P_{\mathrm{ov}}\approx 0$ when ${\mathrm{It}}_{\mathrm{avg}}$ falls roughly 0.5 iterations below ${\mathrm{It}}_{\mathrm{bgt}}$. Thus, the threshold that indicates whether or not the system is overloaded is essentially

(7) $${\mathrm{It}}_{\mathrm{avg}}\overset{\mathrm{overloaded}}{\underset{\mathrm{underloaded}}{≷}}{\mathrm{It}}_{\mathrm{bgt}}.$$

which is what we have used in the above discussion. A similar observation was made in ([26], Figure 8).

When the system is underloaded, each code word is still budgeted a *minimum* of ${\mathrm{It}}_{\mathrm{bgt}}$ iterations, but any of these that are not needed can be transferred to another code word. The *maximum* number of iterations, ${\mathrm{It}}_{\max}$, is not fixed in this case, but rather it fluctuates depending on $L_{\max}$, ${\mathrm{It}}_{\mathrm{bgt}}$, and the receiver operating conditions as characterized by ${\mathrm{It}}_{\mathrm{avg}}$. Because outliers are rare in the underloaded scenario, the typical steady-state assumption is that only one decoder buffer is active, as shown for the beginning and ending time indexes in Figure 17a and Figure 18a.

For β-mode = FALSE, the large red-shaded region in Figure 18a belonging to the outlier (during time indexes 0–7) is easily quantified in terms of $L_{\max}$ and ${\mathrm{It}}_{\mathrm{bgt}}$. Thus, when β-mode = FALSE, the *expected* maximum number of iterations when the system is underloaded is described by the formula

(8) $${\bar{\mathrm{It}}}_{\max}=L_{\max}\left({\mathrm{It}}_{\mathrm{bgt}}-1\right)+1$$

which yields a value of ${\bar{\mathrm{It}}}_{\max}=57$ iterations when fed the parameters of the example in Figure 18.

For β-mode = TRUE, the irregular red-shaded region in Figure 17a belonging to the outlier (during time indexes 0–7) can be described with several terms. When the outlier arrives at time index 0 in Figure 17a, there is a “transient” period where additional decoder buffers are activated (time indexes 0–2); this is a rectangular region that is “tall and skinny” but also missing a triangle at the bottom. If $L_{\max}$ is sufficiently long (as it is in Figure 17), there is an additional red-shaded “steady-state” rectangular-shaped region (time indexes 3–7 in Figure 17). Thus, when β-mode = TRUE, the *expected* maximum number of iterations when the system is underloaded is described by the formula

(9) $${\bar{\mathrm{It}}}_{\max}=\left\{\begin{array}{cc} {\mathrm{It}}_{\mathrm{bgt}}{\mathrm{It}}_{\mathrm{avg}}-\left({\mathrm{It}}_{\mathrm{avg}}-1\right){\mathrm{It}}_{\mathrm{avg}}/2+\left(L_{\max}-{\mathrm{It}}_{\mathrm{avg}}\right)\left({\mathrm{It}}_{\mathrm{bgt}}-{\mathrm{It}}_{\mathrm{avg}}\right)\; & {\mathrm{It}}_{\mathrm{avg}}<L_{\max}\\ {\mathrm{It}}_{\mathrm{bgt}}L_{\max}-\left(L_{\max}-1\right)L_{\max}/2 & \mathrm{otherwise} \end{array}\right.$$

where the various terms in (9) quantify the transient and (possibly) steady-state behavior just described. This formula yields a value of ${\bar{\mathrm{It}}}_{\max}=46$ iterations when fed with the parameters of the example in Figure 17.

## 6. Design Study for LDPC–CPM and Final FER Performance

We now provide examples of a design procedure that is based on the above discussion. This procedure results in a real-time decoder architecture where the maximum number of iterations that need to be performed during a single code word interval has a fixed (and modest) value of ${\mathrm{It}}_{\mathrm{bgt}}$, the maximum decoding latency is fixed at $L_{\max}$ code words, but a relatively large expected maximum value of ${\bar{\mathrm{It}}}_{\max}$ iterations can be allocated to a given code word if needed.

As before, we set our real-time decoder to have a hardware complexity with a fixed budget of ${\mathrm{It}}_{\mathrm{bgt}}=8$ global iterations per code word, and we select the maximum decoding latency to be $L_{\max}=8$ code words (which also determines $N_{\mathrm{buf}}=8$).

Given these design constraints, the real-time decoder (when it is underloaded) can achieve an expected maximum number of global iterations as predicted by (8) or (9), depending on the selection of β-mode. The expected value of ${\bar{\mathrm{It}}}_{\max}$, in turn, results in a predicted FER penalty factor of $X_{512}\left[{\bar{\mathrm{It}}}_{\max}\right]$, relative to a reference operating point. We focus on the P option (puncturing, with ${\mathrm{It}}_{\mathrm{loc}}^{*}$ local iterations) because of its many demonstrated advantages, including effectiveness with smaller (constrained) values of ${\mathrm{It}}_{\max}$. The reference operating points for the six ARTM0 configurations with the P option are listed in Table 2, and Table 3 and Table 4 contain corresponding data for ARTM1 and ARTM2, respectively (18 configurations in total for the P option). We use ${\left(E_{b}/N_{0}\right)}_{\times }$, ${\left(\mathrm{FER}\right)}_{\times }$, and ${\left({\mathrm{It}}_{\mathrm{avg}}\right)}_{\times }$ to denote the numerical values of the reference “×” points plotted in Figure 4, Figure 5 and Figure 6 and Figure 10. All values of ${\left({\mathrm{It}}_{\mathrm{avg}}\right)}_{\times }$ in Table 2, Table 3 and Table 4 are below ${\mathrm{It}}_{\mathrm{bgt}}=8$, and thus the decoder is predicted to be underloaded for any $E_{b}/N_{0}\geq {\left(E_{b}/N_{0}\right)}_{\times }$, although several of the configurations have ${\mathrm{It}}_{\mathrm{avg}}$ that crosses below ${\mathrm{It}}_{\mathrm{bgt}}=8$ at an even earlier point. The values of ${\bar{\mathrm{It}}}_{\max}$ listed in Table 2, Table 3 and Table 4 are used to obtain $X_{512}\left[{\bar{\mathrm{It}}}_{\max}\right]$ via the curves plotted in Figure 12 and Figure 13. The numerical values of the predicted FER penalty from these curves, $X_{512}\left[{\bar{\mathrm{It}}}_{\max}\right]$, are listed in Table 2, Table 3 and Table 4, and are also plotted as the vertical black lines (bars) in Figure 12 and Figure 13.

**Figure 19:**
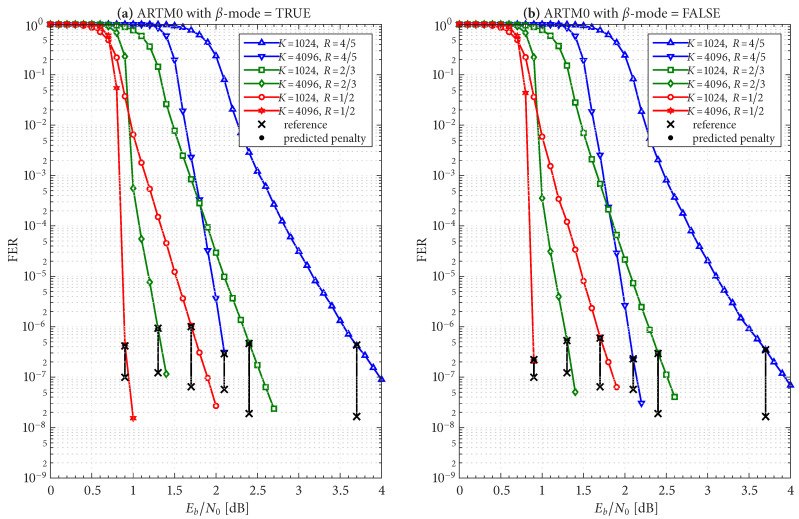
FER curves for the real-time decoder paired with ARTM0 (see also Table 2) for (**a**) β-mode = TRUE and (**b**) β-mode = FALSE.

**Figure 20:**
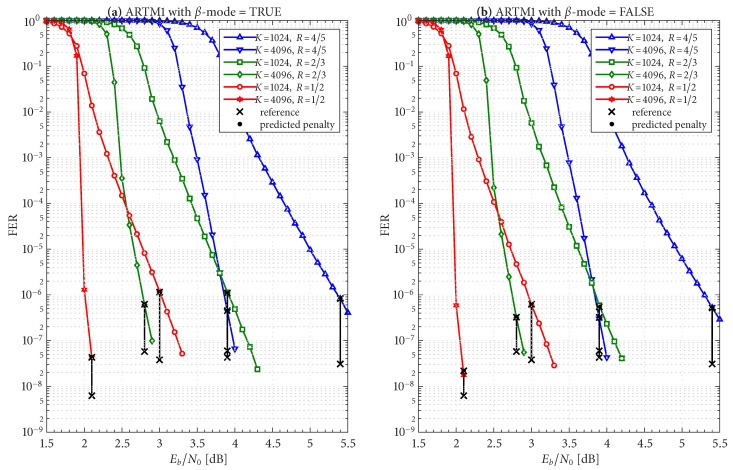
FER curves for the real-time decoder paired with ARTM1 (see also Table 3) for (**a**) β-mode = TRUE and (**b**) β-mode = FALSE.

**Figure 21:**
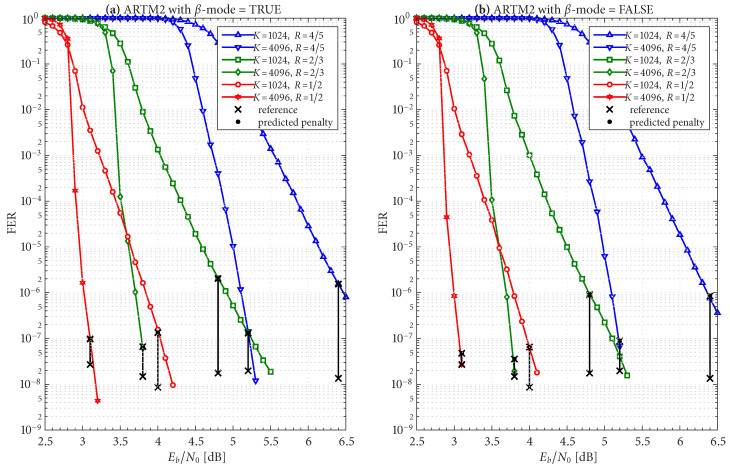
FER curves for the real-time decoder paired with ARTM2 (see also Table 4) for (**a**) β-mode = TRUE and (**b**) β-mode = FALSE.

Figure 19, Figure 20 and Figure 21 show FER simulations of the real-time decoder, respectively, for ARTM0, ARTM1, and ARTM2; each of these have sub-figure (a) giving results for β-mode = TRUE and sub-figure (b) for β-mode = FALSE. As predicted, the decoder is underloaded when $E_{b}/N_{0}$ equals ${\left(E_{b}/N_{0}\right)}_{\times }$. However, the transition between the overloaded/underloaded condition is quite dramatic in some cases. For example, with the K=4096, R=1/2 configurations in Figure 19 and Figure 20, the FER diminishes by *four orders of magnitude* during the final 0.1 dB increment in SNR to reach ${\left(E_{b}/N_{0}\right)}_{\times }$. It is during this narrow SNR interval when ${\mathrm{It}}_{\mathrm{avg}}$ falls below ${\mathrm{It}}_{\mathrm{bgt}}=8$ and a sudden “step up” in maximum iterations comes into effect (i.e., (8) and (9) predict large values of ${\bar{\mathrm{It}}}_{\max}$ in these configurations when underloaded, vs. a maximum of eight iterations when overloaded). The dramatic “step up” in maximum iterations results in the dramatic “step down” in FER that is observed.

These dramatic transitions are also the points where β-mode = FALSE has the highest likelihood of producing “double errors”. At ${\left(E_{b}/N_{0}\right)}_{\times }$ for the K=4096, R=1/2 configurations in Figure 19 and Figure 20, the factor $(1+E\left[{\mathrm{It}}_{\mathrm{bgt}}+1\right]/N_{\mathrm{tot}})\approx 1.10$ predicts a 10% increase in FER, which rapidly goes to 0% as $E_{b}/N_{0}$ continues to increase. However, comparing the values of $X_{512}\left[{\bar{\mathrm{It}}}_{\max}\right]$ in Table 2, Table 3 and Table 4 for β-mode = TRUE vs. β-mode = FALSE, we see that β-mode = FALSE can reduce the FER by as much as 50% relative to β-mode = TRUE, which confirms that it is the superior option between the two.

The black bars in Figure 12 and Figure 13 that predict the FER penalty are copied into Figure 19, Figure 20 and Figure 21, and the base (bottom) of each bar is positioned at its respective reference operating point [see Table 2, Table 3 and Table 4]. In all cases, the top of the black bar (i.e., FER penalty that was predicted in the above design procedure) agrees with the FER that was observed in the simulation of the real-time decoder, for both selections of β-mode. This verifies that a *reference simulation* (such as Figure 4, Figure 5 and Figure 6), conducted using a value of ${\mathrm{It}}_{\max}^{*}$ that is sufficiently large, can be used to quantify the FER performance of a particular configuration of the real-time decoder.

The predicted FER penalties, $X_{512}\left[{\bar{\mathrm{It}}}_{\max}\right]$, listed in Table 2, Table 3 and Table 4, appear to be large. However, when placed in the context of the FER simulations in Figure 19, Figure 20 and Figure 21, we are able to translate these into *SNR penalties*, denoted as ${\left(E_{b}/N_{0}\right)}_{\Delta }$, which are more meaningful. The vertical black bars are helpful in quantifying ${\left(E_{b}/N_{0}\right)}_{\Delta }$, which is defined as the amount of *additional* $E_{b}/N_{0}$ required in order for the respective FER curve of the real-time decoder to reach the bottom of its reference black bar. Table 2, Table 3 and Table 4 list the value of ${\left(E_{b}/N_{0}\right)}_{\Delta }$ measured at the respective operating points for both selections of β-mode.

In cases where the slope of the FER curve is steep (such as the K=4096, R=1/2 configurations already discussed in some detail), we observe ${\left(E_{b}/N_{0}\right)}_{\Delta }<0.1$ dB. However, the K=1024, R=4/5 configurations have a much shallower FER slope, which translates to values of ${\left(E_{b}/N_{0}\right)}_{\Delta }$ approaching one dB; this is also true for the K=1024, R=2/3 configuration for ARTM2. As with other challenges facing these configurations, the smaller value of ${\mathrm{It}}_{\mathrm{loc}}^{*}$ is to blame for these larger FER penalties.

In these cases, the FER penalty can be reduced by increasing ${\bar{\mathrm{It}}}_{\max}$, and as (8) indicates, this is accomplished by increasing ${\mathrm{It}}_{\mathrm{bgt}}$ or $L_{\max}$ as hardware and operational constraints allow. The accuracy of our design methodology means that the FER performance of this parameter space can be explored without the need for additional FER simulations.

## 7. Design Comments for AR4JA–ARTM1-NR

The real-time iterative chain in Figure 16 can be readily adapted to an LDPC decoder iterating by itself, which is the case for AR4JA–ARTM1-NR. The storage requirements of the memory buffers are simplified by the absence of the CPM SISO decoder.

The status of such a decoder being underloaded and overloaded is once again decided by (7), and thus the reference values of ${\mathrm{It}}_{\mathrm{avg}}$ in Figure 10 are of particular interest. For the K=4096, R=1/2 AR4JA–ARTM1-NR decoder, a relatively large budget of ${\mathrm{It}}_{\mathrm{bgt}}\approx 20$ is required (orange curve in Figure 10e). Using (8), a latency of $L_{\max}=4$ is sufficient to achieve ${\bar{\mathrm{It}}}_{\max}=77$ iterations, and judging by the orange FER penalty factor curve in Figure 12c, this number of iterations would result in negligible performance losses. Thus, the reference data in Figure 10, Figure 12 and Figure 13 are sufficient to understand the complexity budget and decoding latency that are needed in order to realize acceptably small performance losses.

## 8. Conclusions

In this paper, we have presented comprehensive reference results that characterize the FER performance and iteration statistics of three example LDPC-CPM schemes and a related LDPC-only scheme; in particular, we quantified the large “peak to average” ratio of global iterations in these LDPC-based systems. A significant initial finding is that there are a number of advantages for a decoder configuration that performs a few local (LDPC) iterations per global decoding iteration. We showed how the iteration statistics drawn from a single reference simulation with a large value of maximum iterations can be used to characterize the FER performance of any simulation that is configured with a smaller value of maximum iterations. We then developed a real-time decoder architecture that performs a fixed and relatively small number of global iterations during a single code word interval. We showed how such a decoder can still achieve a relatively large maximum number of global iterations by introducing a trade-off between decoding latency and maximum global iterations. We presented a comprehensive design example that demonstrated the accuracy of our methodology in predicting the performance of the real-time decoder. We also showed that the real-time decoder can operate with modest complexity, with accompanying SNR penalties of less than a tenth of a dB. And finally, although aeronautical telemetry and LDPC–CPM are the main focus of our work, we showed how these results are generally applicable to other LDPC-based systems.

## Figures and Tables

**Table 1:** Dimensions of the final LDPC codes.

*K*	*R*	$N=N_{\mathrm{NP}}$	${\mathrm{It}}_{\mathrm{loc}}^{*}$	$N_{P}$
1024	4/5	1280	1	1344
1024	2/3	1536	2	1664
1024	1/2	2048	4	2304
4096	4/5	5120	4	5376
4096	2/3	6144	4	6656
4096	1/2	8192	4	9216

**Table 2:** Final design values for ARTM0.

					β-Mode = TRUE			β-Mode = FALSE		
K	R	(*E_b_*/*N*_0_)_×_	(FER)_×_	(*It*_avg_)_×_	${\bar{\mathrm{It}}}_{\max}$	*X*_512_[${\bar{\mathrm{It}}}_{\max}$]	(*E_b_*/*N*_0_)_Δ_	${\bar{\mathrm{It}}}_{\max}$	*X*_512_[${\bar{\mathrm{It}}}_{\max}$]	(*E_b_*/*N*_0_)_Δ_
1024	4/5	3.7 dB	$1.6\;\times \;10^{-8}$	2.9	46.4	26.3	0.6 dB	57	21.1	0.4 dB
1024	2/3	2.4 dB	$1.9\;\times \;10^{-8}$	3.5	43.9	24.6	0.3 dB	57	15.6	0.3 dB
1024	1/2	1.7 dB	$6.4\;\times \;10^{-8}$	4.1	41.7	15.6	0.2 dB	57	9.3	0.2 dB
4096	4/5	2.1 dB	$5.7\;\times \;10^{-8}$	3.7	43.0	5.1	<0.1 dB	57	4.1	<0.1 dB
4096	2/3	1.3 dB	$12.2\;\times \;10^{-8}$	5.4	38.0	7.7	<0.1 dB	57	4.4	<0.1 dB
4096	1/2	0.9 dB	$9.9\;\times \;10^{-8}$	7.2	35.9	4.2	<0.1 dB	57	2.2	<0.1 dB

**Table 3:** Final design values for ARTM1.

					β-Mode = TRUE			β-Mode = FALSE		
K	R	(*E_b_*/*N*_0_)_×_	(FER)_×_	(*It*_avg_)_×_	${\bar{\mathrm{It}}}_{\max}$	*X*_512_[${\bar{\mathrm{It}}}_{\max}$]	(*E_b_*/*N*_0_)_Δ_	${\bar{\mathrm{It}}}_{\max}$	*X*_512_[${\bar{\mathrm{It}}}_{\max}$]	(*E_b_*/*N*_0_)_Δ_
1024	4/5	5.4 dB	$3.1\;\times \;10^{-8}$	3.2	45.2	26.9	0.6 dB	57	16.8	0.5 dB
1024	2/3	3.9 dB	$4.3\;\times \;10^{-8}$	3.8	42.9	25.4	0.3 dB	57	12.8	0.2 dB
1024	1/2	3.0 dB	$3.8\;\times \;10^{-8}$	4.0	42.0	30.2	0.3 dB	57	16.5	0.2 dB
4096	4/5	3.9 dB	$6.0\;\times \;10^{-8}$	3.6	43.4	7.6	0.1 dB	57	5.4	<0.1 dB
4096	2/3	2.8 dB	$5.8\;\times \;10^{-8}$	5.3	38.2	10.8	0.1 dB	57	5.6	<0.1 dB
4096	1/2	2.1 dB	$0.6\;\times \;10^{-8}$	6.9	36.1	6.8	<0.1 dB	57	3.5	<0.1 dB

**Table 4:** Final design values for ARTM2.

					β-Mode = TRUE			β-Mode = FALSE		
K	R	(*E_b_*/*N*_0_)_×_	(FER)_×_	(*It*_avg_)_×_	${\bar{\mathrm{It}}}_{\max}$	*X*_512_[${\bar{\mathrm{It}}}_{\max}$]	(*E_b_*/*N*_0_)_Δ_	${\bar{\mathrm{It}}}_{\max}$	*X*_512_[${\bar{\mathrm{It}}}_{\max}$]	(*E_b_*/*N*_0_)_Δ_
1024	4/5	6.4 dB	$1.3\;\times \;10^{-8}$	3.1	45.6	114.7	0.7 dB	57	63.5	0.6 dB
1024	2/3	4.8 dB	$1.7\;\times \;10^{-8}$	4.1	41.8	116.4	0.7 dB	57	51.5	0.5 dB
1024	1/2	4.0 dB	$0.9\;\times \;10^{-8}$	4.2	41.5	15.5	0.2 dB	57	7.5	0.1 dB
4096	4/5	5.2 dB	$2.0\;\times \;10^{-8}$	3.7	43.1	6.7	<0.1 dB	57	4.5	<0.1 dB
4096	2/3	3.8 dB	$1.5\;\times \;10^{-8}$	5.7	37.4	4.5	<0.1 dB	57	2.4	<0.1 dB
4096	1/2	3.1 dB	$2.7\;\times \;10^{-8}$	6.8	36.2	3.6	<0.1 dB	57	1.8	<0.1 dB

## Data Availability

The original contributions presented in the study are included in the article, further inquiries can be directed to the corresponding author.

## Distribution Statement

Approved for public release; Distribution is unlimited 412TW-PA-24182.
